# Subglacial discharge controls seasonal variations in the thermal structure of a glacial lake in Patagonia

**DOI:** 10.1038/s41467-021-26578-0

**Published:** 2021-11-02

**Authors:** Shin Sugiyama, Masahiro Minowa, Yasushi Fukamachi, Shuntaro Hata, Yoshihiro Yamamoto, Tobias Sauter, Christoph Schneider, Marius Schaefer

**Affiliations:** 1 Institute of Low Temperature Science, Hokkaido University, Sapporo, Japan; 2 Arctic Research Center, Hokkaido University, Sapporo, Japan; 3 Graduate School of Environmental Science, Hokkaido University, Sapporo, Japan; 4 Institute of Geography, Friedrich-Alexander-Universität Erlangen-Nürnberg, Erlangen, Germany; 5 Geography Department, Humboldt-Universität zu Berlin, Berlin, Germany; 6 Instituto de Ciencias Físicas y Matemáticas, Universidad Austral de Chile, Valdivia, Chile

**Keywords:** Cryospheric science, Hydrology, Limnology

## Abstract

Water temperature in glacial lakes affects underwater melting and calving of glaciers terminating in lakes. Despite its importance, seasonal lake temperature variations are poorly understood because taking long-term measurements near the front of calving glaciers is challenging. To investigate the thermal structure and its seasonal variations, we performed year-around temperature and current measurement at depths of 58–392 m in Lago Grey, a 410-m-deep glacial lake in Patagonia. The measurement revealed critical impacts of subglacial discharge on the lake thermal condition. Water below a depth of ~100 m showed the coldest temperature in mid-summer, under the influence of glacial discharge, whereas temperature in the upper layer followed a seasonal variation of air temperature. The boundary of the lower and upper layers was controlled by the depth of a sill which blocks outflow of dense and cold glacial meltwater. Our data implies that subglacial discharge and bathymetry dictate mass loss and the retreat of lake-terminating glaciers. The cold lakewater hinders underwater melting and facilitates formation of a floating terminus.

## Abstract (web summary)

Thermal conditions and circulation near glacier fronts are important to understand the recent rapid retreat of calving glaciers. New observations from a glacial lake suggesting a feedback mechanism between atmospheric warming, glacier front melting and calving for freshwater-terminating glaciers.

## Introduction

Glaciers terminating in water change more rapidly than those terminating on land^1,2^. Rapid changes of water-terminating glaciers are affected by underwater frontal melting, which in turn destabilizes the ice front and generates calving^3,4^. The importance of underwater melting and its influence on calving has been recognized recently based on oceanic measurement near the front of marine-terminating glaciers^5–8^. In contrast to intensive research in marine settings, studies on freshwater calving glaciers and glacial lakes are sparse. Mass loss of freshwater calving glaciers is important in Patagonia^9^, Alaska^10^, New Zealand^11^, and the Himalayas^12^, among other regions. The importance we place on freshwater calving is increasing, because glacial lakes are newly formed at the forefront of retreating glaciers^13,14^. Moreover, proglacial lakes played a significant role in the deglaciation of the former continental ice sheets^15^, and thus the inclusion of lake processes is required in numerical models^16^. Therefore, physical conditions of glacial lakes are needed to understand the rapid changes of freshwater calving glaciers in the past, present, and future^4,17^.

Water circulation in lakes in contact with glaciers differs significantly from that in glacial fjords. Near the front of a marine-terminating glacier, subglacial discharge causes an upwelling plume because of the difference in density between glacial meltwater and seawater^4,18,19^. Relatively warm, deep ocean water entrained by the plume upwells along the glacier front and supplies heat for melting. However, near the front of freshwater calving glaciers, no evidence has been reported for upwelling and active circulation. This lack of circulation affects the lake thermal regime and underwater melting. Measurements of glacial lakes in Alaska^20,21^, New Zealand^22,23^, Himalaya^24,25^, and Patagonia^26,27^ showed relatively cold stratified water, implying insignificant underwater melting. A comprehensive study of three glacial lakes in Patagonia reported an absence of upwelling, but instead confirmed that subglacial discharge sinks deep into a lake because glacial meltwater is turbid and thus denser than lakewater^27^. Temperature and turbidity measurements indicated stratification of lakewater into a relatively cold/turbid lower layer and a warm/clear upper layer. Because the stratification prohibits vertical mixing, atmospheric heat absorbed by surface water is distributed only within the upper layer and contributed to ice-front melting near the lake surface. Enhanced melting near the lake surface leads to a protrusion of the deeper ice into the water, which was observed by a side-scan sonar survey as an “underwater ice terrace” at the front of Glaciar Grey^28^. The submerged ice destabilizes the glacier front by exerting a buoyant force, thus the observed ice front geometry provided an important implication for buoyancy-driven underwater calving. These observations demonstrated that the stratification of a glacial lake dictates the calving mechanism and frontal dynamics of glaciers terminating in the lake.

Previous studies have measured and examined the summer conditions of glacial lakes^20–28^. Nevertheless, the water temperature and circulation of these lakes are subjected to seasonal variations in both atmospheric conditions and subglacial discharge. Since long-term measurements are difficult to obtain near calving fronts, year-around in-situ temperature data have thus far never been reported. To investigate seasonal variations in the thermal structure of a glacial lake, we obtained temperature and current measurements by installing a mooring in Lago Grey, a proglacial lake of Glaciar Grey in the Southern Patagonia Icefield. Observations over a period of 20 months revealed strong influences of subglacial discharge on the lake thermal structure.

## Results

### Study site

**Fig. 1:**
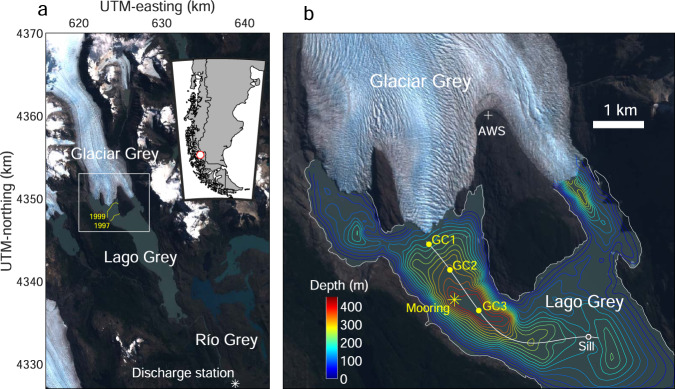
Study site. **a** Satellite image of Glaciar Grey and Lago Grey (Landsat 8, 4 February 2017; UTM zone 18S). Inset and box show the location of the region in South America and the area covered by (**b**), respectively. The yellow curves show the glacier front positions in March 1997 and February 1999 as mapped by Landsat 5 images. The Río Grey discharge station is indicated by (✳). **b** Bathymetry of Lago Grey shown by contour lines with intervals of 25 m. Markers indicate the locations of the mooring (✳), temperature and turbidity profiler casting (●), automatic weather station (+), and sill mentioned in the text (○). The solid line shows the locations of the cross-section in Fig. 2e.

Lake measurements were obtained in Lago Grey, a glacial lake on the southeastern edge of the Southern Patagonia Icefield (51.0°S, 73.2°W) (Fig. 1a). The lake is situated along a glaciated valley with a length of 16 km and an area of 38.0 km^2^ in 2017. Glaciar Grey, a freshwater calving glacier, feeds the lake from the north. Lakewater then drains to the south into its outlet stream, Río Grey. Glaciar Grey covers an area of 243 km^2^ over an elevation range of 60–2360 m a.s.l. (ref. ^29^). After a long-lasting retreat during the 20th century^30^, the glacier front has been relatively stable in the 21st century. The glacier currently flows into the lake through three separated termini, seeing as the terminus separated into central and western parts during a rapid retreat in the late 1990s. The rapid retreat was attributed to the overdeepening of the bed geometry^30^, but to date lake bathymetry has only been reported for the region near the eastern terminus^28^. Our measurements took place in the over-deepened region near the central glacier terminus, which flows at a rate of ~1 km a^−1^ (refs. ^31,32^) over a ~1000-m wide calving front (Fig. 1b).

### Summer water temperature structure

**Fig. 2:**
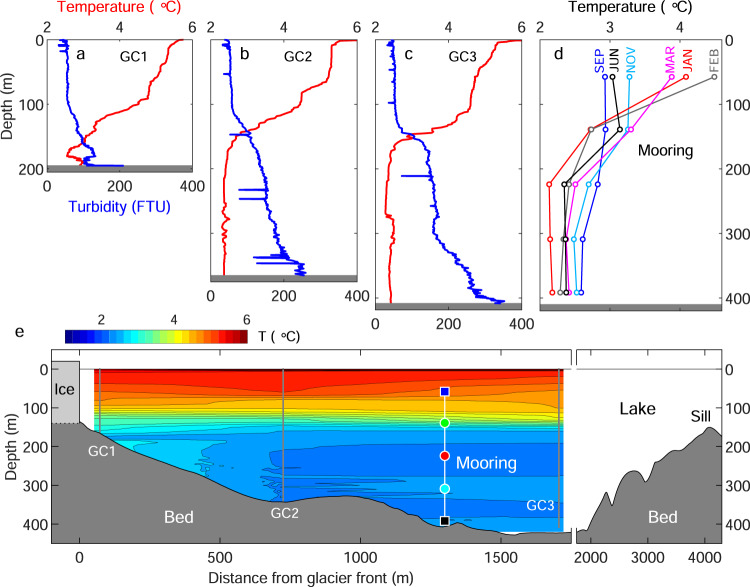
Lakewater temperature and turbidity in Lago Grey on 10 March 2017. **a**–**c** Water temperature (red) and turbidity (blue) profiles obtained at the sites GC1–3 (Fig. 1b). **d** Monthly mean temperature profiles obtained by the mooring sensors. **e** Water temperature and bed elevation within a cross-section along the solid line in Fig. 1b. Contour lines with intervals of 0.2 °C are obtained by linear interpolation of the temperature profiles observed at GC1–3. Markers show the depths of the temperature (●) and current/temperature sensors (■), and their approximate distance from the glacier. Marker colors correspond to line colors in Figs. 3b and 6e–h. Note a change in the x-axis scale.

In March 2017, lakewater showed a stratification with a warmer upper layer and a colder lower layer (Fig. 2). Between 100 and 1700 m from the glacier front (GC1–3 in Fig. 1b), water temperature in the upper 100 m was warmer than 4 °C (red curves in Fig. 2a–c). A sharp drop in temperature was observed at each of the three casting sites within a layer approximately 100–150 m deep. The temperature was uniformly distributed below the depth of 200 m (2.3–2.5 °C), where mean temperatures were 2.37 °C at GC2 and 2.39 °C at GC3.

The vertical temperature distributions were associated with turbidity profiles (blue curves in Fig. 2a–c). Turbidity was low and fairly uniform above the depth of 100 m (~50 FTU). Water became more turbid below the depths of 100–150 m, corresponding to the observed thermocline. Turbidity gradually increased in the deeper region and a greater increase was observed near the bottom (~300–400 m) at GC2 and GC3.

Overall, the water was relatively warm and clear in the upper 100 m, with colder and more turbid water below the depth of ~150 m. The boundary of the two layers approximately coincided with the bed elevation of a sill located 4 km from the glacier front (Figs. 1b and 2e).

### Seasonal water temperature variations

**Fig. 3:**
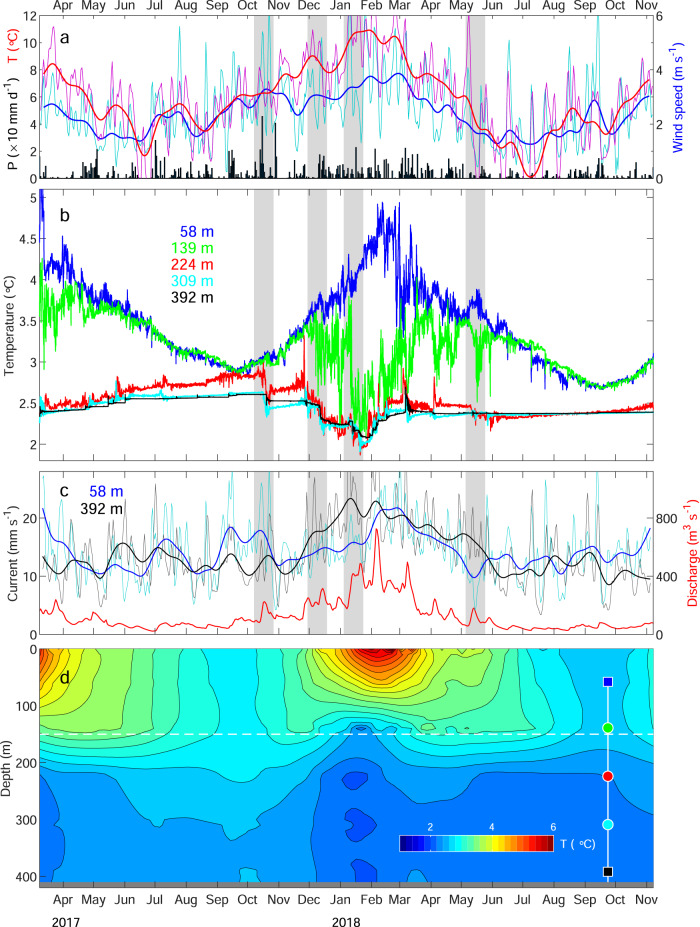
Atmospheric, lake, and river observations from March 2017 to November 2018. **a** Air temperature (red), wind speed (blue), and daily liquid precipitation (black) obtained at the weather station (Fig. 1b). Hourly temperature and wind speed were filtered with 1-d (thin) and 1-week windows (thick). Grey shadings are time periods shown in Fig. 6. **b** Water temperature measured by mooring at the depths of 58 (blue), 139 (green), 224 (red), 309 (cyan), and 392 m (black) (Figs. 1b and 2e). **c** Current speeds measured by mooring at the depths of 58 (blue) and 392 m (black), and discharge of Río Grey (red) recorded at the station indicated in Fig. 1a. Currents were filtered with 1-d (thin) and 1-week windows (thick). **d** Water temperature distribution obtained by interpolating the data in (**b**). The temperature was linearly extrapolated to the regions above 58 m and below 392 m. Contour intervals are 0.2 °C. The horizontal dashed line indicates the depth of the sill shown in Figs. 1b and 2e. Markers show the depths of the temperature (●) and current/temperature sensors (■) with colors corresponding to the line colors in (**b**) and Fig. 6e–h.

Seasonal variations in the lake conditions were revealed by five temperature sensors and two current meters installed at depths of 58‒392 m at 1.4 km from the glacier front (Figs. 1b and 2e). After the installation in March 2017, the upper two temperature sensors (58 and 139 m) showed cooling until minimal temperatures (2.8 °C) reached in September (Fig. 3b). The timing of the minimal water temperature was about three months later than the coldest air temperature (Fig. 3a), which was recorded at an automatic weather station (AWS) near the glacier front (Fig. 1b). The uppermost sensor subsequently showed warming to the peak temperature of ~5 °C in February 2018, which was followed by cooling towards winter. The maximum water temperature lagged several weeks behind the peaks in air temperature and the discharge of Río Grey (Fig. 3c) measured at 14 km downstream from Lago Grey (Fig. 1a). In contrast to the straightforward seasonal pattern obtained at the top sensor, the temperature at the depth of 139 m began oscillating significantly in December 2017 and sharply dropped to the minimum temperature of 2.1 °C in January 2018 (green curve in Fig. 3b). Thereafter, the temperature gradually increased with high-frequency oscillations and asymptotically approached to the value of the uppermost sensor towards the winter season. The drop in temperature during summer was commonly observed by the three other deeper sensors at 224 − 392 m. In October 2017, a relatively small (~0.1‒0.3 °C) but clear drop was simultaneously observed at the three sensors (Fig. 3b). These sensors indicated further cooling from late November 2017 to January 2018 until temperature then began to increase in late January and progressively increased in February. Temperatures were relatively stable after late February, except for short episodes of warming in March and April.

### Seasonal current variations

Current speed was highly variable over a range of 4‒36 mm s^−1^ (after filtering with a 1-d window) near the surface (58 m) and 3‒40 mm s^−1^ near the bottom (392 m) (Fig. 3c). Summer mean speeds (December 2017 to February 2018) were 17 and 21 mm s^−1^ for the upper and lower sensors, respectively, which were 10 and 40% greater than the annual mean (July 2017 to June 2018, 15 mm s^−1^ for both sensors). The upper sensor peaked in February–March, lagging approximately one month behind the lower sensor.

**Fig. 4:**
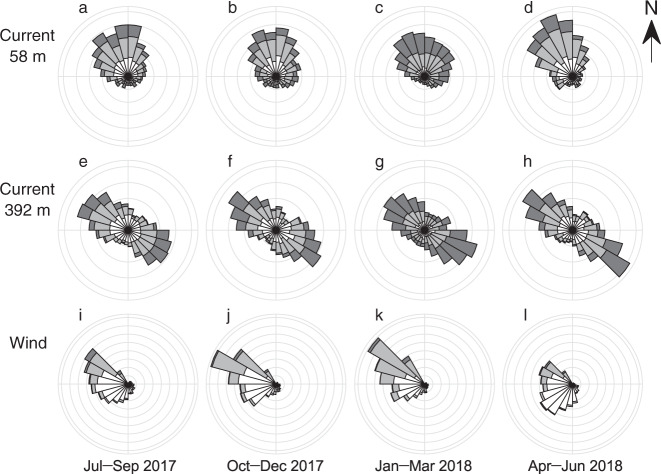
Wind and current directions. Water current direction at the depths of (**a**)–(**d**) 58 m and (**e**)–(**h**) 392 m, for July–September 2017, October–December 2017, January–March 2018, and April–June 2018. White, light grey, and dark grey in (**a**)–(**h**) indicate the current speed of <10, 10–20, and >20 mm s^‒1^. (**i**)–(**l**) are the same for wind speed. White, light, and dark grey in (**i**)–(**l**) indicate wind speed of <5, 5–10, and >10 m s^‒1^.

Although annual mean speeds were similar, current directions were significantly different at the two sensors. Flow at the upper sensor was dominated by north-northwesterly current (Fig. 4a–d). The annual mean current direction was 333°, which approximately coincides with the azimuth of the glacier front (340°) as well as the northwesterly prevailing wind direction observed at the AWS (Fig. 4i–l). Flow at the lower sensor aligned northwest-southeast, approximately along the trough of the lake (Fig. 4e–h). Flow directions were evenly distributed to the northwest and southeast, thus net water transport was small near the bottom. Neither the upper nor lower sensor showed significant seasonal variations in the flow direction.

**Fig. 5:**
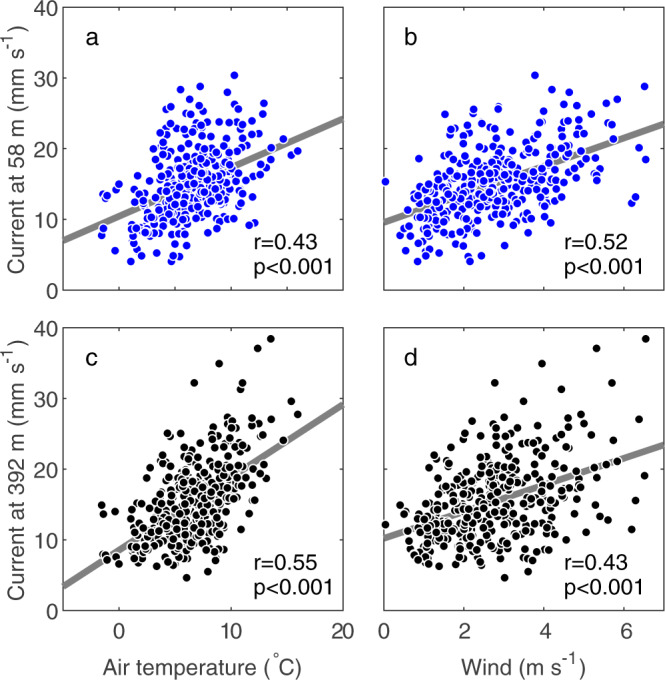
Correlations between current and AWS data. Scatter plots of daily current speed at the depth of 58 m versus (**a**) air temperature and (**b**) wind speed from July 2017 to 2018. (**c**) and (**d**) are the same plots for current at 392 m. Current and wind speeds were filtered with a 1-d window. The grey lines are a linear regression of the data with Pearson correlation coefficients *r* and *p*-values.

Water current was stronger in summer when AWS data showed greater wind speed as well as higher air temperature (Fig. 3a and c). The speed at a depth of 58 m showed a higher correlation with wind speed (Pearson correlation coefficient: *r* = 0.52, *p*-value: *p* < 0.001) than with air temperature (*r* = 0.43, *p* < 0.001) (Fig. 5a, b). At a depth of 392 m, however, the correlation coefficient with air temperature (*r* = 0.55, *p* < 0.001) was greater than that with wind speed (*r* = 0.43, *p* < 0.001) (Fig. 5c, d). These statistics suggest that the magnitude of the near surface water current was related to wind, whereas the strong current in the deeper regions was linked to a warm atmospheric condition.

## Discussion

The stratification of Lago Grey observed in summer, i.e. warm/clear water above cold/turbid water, is consistent with observations made in other glacial lakes in Patagonia^27^. Turbid subglacial discharge fills the depth below the 150-m-deep sill, whereas atmospheric heating and wind-driven circulation generate the warm upper layer (Fig. 2e). Here, we provide interpretations of the temperature and current data obtained using this mooring measurement, which reveals seasonal variations in the thermal structure and circulation of a glacial lake in Patagonia.

### Interpretation of the data

**Fig. 6:**
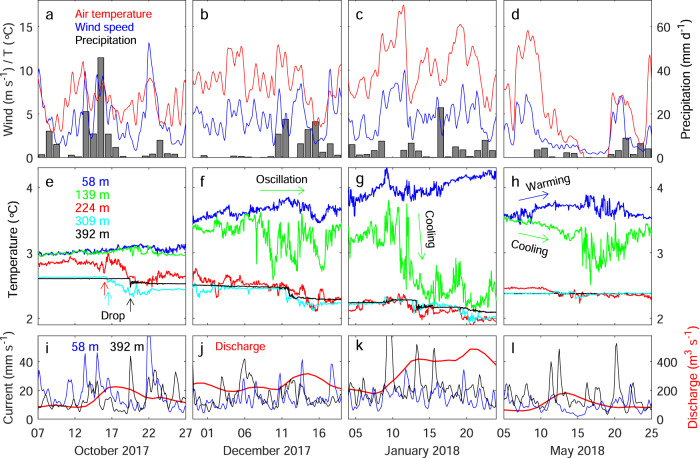
Observations for the periods highlighted in Fig. 3. **a**–**d** Air temperature (red), wind speed (blue), and daily liquid precipitation (black). Temperature and wind speed were filtered with a 3-h window. **e**–**h** Water temperature at depths of 58 (blue), 139 (green), 224 (red), 309 (cyan), and 392 m (black). **i**–**l** Current speeds at the depths of 58 (blue) and 392 m (black) filtered with a 3-h window, and the discharge of Río Grey (red).

Temperature from the lower four sensors (139–392 m) dropped during the summer (Fig. 3b), suggesting an impact of glacial meltwater discharge. In summer 2017/18, the first clear temperature drop was observed on 16 October 2017 at depths of 224 and 309 m (Fig. 6e). The lowest sensor (392 m) showed a smaller change three days later, whereas no clear change was observed at the upper two sensors. The drop in temperature was preceded by a rain event that began on 13 October 2017 (Fig. 6a). The daily precipitation on 15 October (46 mm d^−1^) was the largest over the entire study period and the cumulative precipitation from 13–16 October was 88 mm. The air temperature during the event (~5–10 °C) suggests that liquid precipitation fell over a large portion of the ablation area. The rain event affected the river discharge, which increased from 82 to 225 m^3^ s^−1^ during 12–17 October (Fig. 6i). Presumably, a large amount of rainwater drained as subglacial discharge and caused the temperature change in the deeper region. Current at the lower sensor (392 m) increased to >40 mm s^−1^ on 19 October when the temperature dropped at the same depth (black curves in Fig. 6e and i). The upper current sensor (58 m) peaked several times in October, which coincided with strong wind events (blue curves in Fig. 6a and i). We assume that the currents in near-surface water were generated by wind stress as suggested by the correlation analysis (Fig. 5b).

The temperature at the second top sensor (139 m) began oscillating in December (green curve in Fig. 6f). The oscillation was associated with gradual cooling at the deeper three sensors. Around the onset of the oscillation, air temperature indicated a long spell of warm weather (Fig. 6b), and current at the deeper sensor increased on 5–6 December (black curve in Fig. 6j). Monthly mean river discharge increased by 65% from November to December 2017 (Fig. 3c). We assume that an increasing amount of meltwater drained into the lake as subglacial discharge during this period, affecting the mid-depth water as well as the deeper region. Meltwater production under a warm atmospheric condition, and its discharge into the lake bottom, explain the relatively high correlation between air temperature and currents in deep water (Fig. 6c). The peaks in the upper current sensor on 10, 13, and 17 December are attributed to strong wind (blue curves in Fig. 6b and j) rather than the air temperature.

The temperature at the second sensor (139 m) dropped by >1 °C from 10 to 12 January 2018 (green curve in Fig. 6g). This event occurred during especially warm atmospheric conditions, represented by the highest hourly air temperature over the study period (19.4 °C on 11 January) (Fig. 6c). The period was also characterized by a strong current near the lake bottom, including the fastest speed over the entire period on 9 January (118 mm s^−1^ after filtering with a 3 h window) (black curve in Fig. 6k). Most likely, intensive glacier melting resulted in a great amount of subglacial discharge into the lake, which is consistent with a twofold increase in river discharge from 8–13 January (Fig. 6k). The temperature change at 139 m implies that the layer below the sill depth (150 m) had been filled with glacial meltwater by that time. Upward migration of the thermocline affected the region shallower than the sill, as depicted in Fig. 3d. The cold bottom layer (<~2.5 °C) gradually thickened from October 2017, reaching maximum thickness in January–February 2018 (Fig. 3d).

After mid-summer, the upper two sensors occasionally exhibited contrary temperature change, i.e. near-surface warming and sub-surface cooling. In May, for instance, the second sensor showed ~1 °C cooling while temperature increased at the top sensor (Fig. 6h). The deviation of the temperatures was preceded by a period of warm air temperature (Fig. 6d), suggesting the influence of glacial discharge on the sub-surface layer and atmospheric heating near the surface. The temperature dropped as well at the lower two sensors (224 and 309 m), but the change was greater at 139 m. Presumably, subglacial discharge directly affected the mid-depth layer because turbid water had already occupied the deeper regions and blocked the submergence of subglacial discharge.

### Seasonal variations in lake thermal structure

**Fig. 7:**
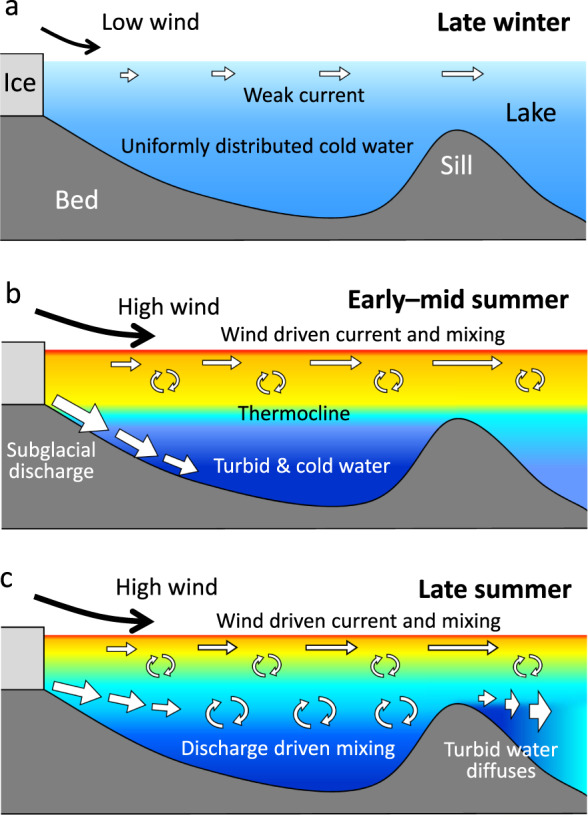
Schematic diagrams showing seasonal variations in lake thermal structures and circulation. **a** The lake in late winter is characterized by weak current and uniformly distributed relatively cold water. **b** In early-mid summer, cold and dense subglacial discharge fills the lake deeper than the sill. Atmospheric heat is distributed to the upper layer by wind-driven circulation, resulting in the formation of a thermocline at the sill depth. **c** Subglacial discharge in later summer jets into the mid-layer, which drives mixing of upper warm and lowers cold waters.

Based on the observations described above, we can summarize the seasonal variations in the thermal structure of Lago Grey. In September, water temperature fell in a narrow range (2.5–3 °C) with a minimal vertical gradient (Fig. 2d). The wind is relatively weak in winter, thus circulation in the lake is insignificant (Fig. 7a). The temperature subsequently increased in the upper ~100 m and reached the annual maximum in February (blue curve in Fig. 3b). The heat from the atmosphere and solar radiation was distributed to the near-surface layer through mixing generated by a prevailing northwesterly wind (Fig. 7b). In contrast to the upper layer, the temperature dropped below the depth of ~150 m from September to January (Figs. 2d and 3b). This was due to subglacial discharge, which generates a cold and turbid lower layer gradually thickening towards mid-summer (Fig. 3d). The depth of the upper-lower layer boundary was controlled by the elevation of the sill, which acted as a dam of dense and cold glacial meltwater (Figs. 2e and 7b). Water temperature in the lower layer began to increase in January during the midst of melt season (Figs. 2d and 3b). We hypothesize that after mid-summer, subglacial discharge was directly injected into the mid-depths of the lake and induced vertical mixing with the warmer upper layer (Fig. 7c). Sediment concentration in subglacial discharge is greater in early summer during the development of the subglacial drainage system and it progressively decreases later in the melt season^33,34^. Thus, density of the subglacial discharge is expected to decrease over the course of summer. Presumably, meltwater jetting at the depth of ~150 m flowed over the more turbid deep water originating from early summer discharge. The existence of the most turbid deep water is supported by the turbidity measurement in March (Fig. 2b, c).

### Implication for glacier front melting, calving, and lake ecosystem

The year-round temperature data indicates that lakewater is consistently cold, except for the upper ~150 m, which is affected by heat from the atmosphere during summer. The temperature below 200 m is colder than 3 °C throughout the year under the influence of subglacial discharge (Figs. 2d and 3d), suggesting relatively small rates of underwater melting. The upper-most layer (<~50 m deep) is above 4 °C from January to March, implying that the most significant melting occurs during this period. Under this condition, ice melts only near the water surface and deeper sections tend to project into the lake as reported in previous studies^28,35^, which reduces frequency and rate of calving as compared to marine-terminating glaciers in the same geometrical settings^2,3^.

Our data also demonstrate the importance of sill depth for the thermal regime of a glacial lake. The thermocline in Lago Grey is located at the depth of the sill as reported in the proglacial lake of Glaciar Viedma in Patagonia^27^. Coldwater dammed up by the sill reduces melting and preserves ice in the lake, which may lead to the formation and large-scale calving of a floating terminus^4^. In 1997, the terminus of Glaciar Grey situated in overdeepening at our study area disintegrated over an area of 1.5 km^2^ (ref. ^30^) (Fig. 1a). This event was possibly due to a buoyancy force acting on terminus thinned in excess of hydrostatic equilibrium, as reported in Yakutat Glacier in Alaska^36^ and Glaciar Nef in Patagonia^37^. We assume that the depth and location of the sill played a decisive role in the timing and magnitude of the disintegration. Lake bathymetry is therefore critical to understand the rapid retreat of freshwater calving glaciers as well as the dominant mechanism of frontal ablation. Presumably, frontal ablation in deep lakes with a sill is dominated by infrequent large calving driven by buoyancy, whereas underwater melting and frequent smaller calving are more important in shallow lakes.

Our results also carry an implication for the ecosystem near the glacier front. Recent studies in Greenland and Svalbard demonstrated the important role of glacial discharge in marine biology. Upwelling plumes at the front of marine-terminating glaciers facilitate the formation of biological hotspots, by supplying nutrients and plankton to the fjord surface^38,39^. In contrast to the increasing number of studies in glacial fjords, very little is known about the ecosystem of lakes fed by calving glaciers. Observations in Lago Grey indicate that the aquatic conditions near the glacier front are largely different from those reported for marine-terminating glaciers. Presumably, the ponding of cold and turbid water greatly influences the growth, metabolism, reproduction, and foraging of life in the lake. Moreover, changes in the amount of glacial discharge as well as retreat of the glacier give rise to a critical impact on the biological environment of glacial lakes, as predicted for glacial fjords, and glacier-fed river and lake systems^40–42^.

## Methods

### Lake depth

The depth of Lago Grey was measured within several kilometers from the glacier in January and February 2016, March 2017, and November 2018 (Fig. 1b). We operated an ultrasonic echo sounder (Lowrance HDS-7) with a frequency-modulated transducer (Airmar B75M, 80–130 kHz) mounted on a boat. Water depth was recorded every 1 s with horizontal coordinates obtained by a single-positioning GPS integrated in the sounder. The accuracy of the depth measurement was ~5 m or ~5% of the depth, according to the previous measurements with a similar device in Patagonian lakes^27^.

### Seasonal variations in water temperature and current

A mooring was deployed at 1.4 km from the central terminus (Fig. 1b). The site is in the deepest region of the overdeepening, where the lake is 410 m deep according to our sonar sounding. The mooring consisted of three temperature loggers (Seabird, SBE56) at depths of 139, 224, and 309 m from the surface, and two current/temperature loggers (JFE Advantech, INFINITY-EM) at 57.5 and 391.5 m (Fig. 2e). The accuracies of the measurement were 0.002 °C for SBE56, and 10 mm s^−1^, ±2°, and 0.02 °C for INFINITY-EM. The azimuth of current was corrected for the magnetic declination in the region (13.5°E). The measurement was performed with intervals of 1 min (temperature loggers) and 1 h (current/temperature loggers) from 7 March 2017 to 8 November 2018 for 611 days. To retrieve the instruments by disconnecting the mooring from ballast, we used a motor-driven releaser and an acoustic controller (Kaiyo Denshi, TMR-6005B and SCA-10B). Lakewater temperature was processed for hourly mean values. Hourly current speed was filtered by Gaussian smoothing filters with time windows of 3 h, 1 d, or 1 week.

### Depth profiles of water temperature and turbidity

Lakewater temperature and turbidity distributions from the surface to the bottom were measured on 10 March 2017 at 70, 730, and 1710 m from the glacier front (GC1–3 in Fig. 1b). A temperature, turbidity, and depth profiler (JFE Advantech, ASTD101) was lowered from a boat to record the water properties every 1 s (~0.2–1 m in depth). The accuracies of the depth, temperature, and turbidity measurements were ±1.8 m, ±0.01 °C, and ±0.3 FTU (formazin turbidity unit) or 2% of measured turbidity, respectively.

### Meteorology and river discharge

Meteorological measurement was performed by an AWS installed at 229 m a.s.l. on the bedrock between the central and eastern termini^43,44^ (Fig. 1b). Air temperature, liquid precipitation, and wind speed/direction sensors (Campbell Scientific; CS215, 52202-L, and 05108-45-L) were installed at 2, 2.5, and 0.5 m from the ground, and measurements were taken every 10 min. The uncertainties of the temperature, precipitation, and wind measurement were ±0.4 °C, ±3%, and ±0.3 m s^−1^/±3°, respectively. Air temperature and wind speed were filtered by Gaussian smoothing filters with 3-h, 1-d, and 1-week time windows.

We compared our measurements with data from the downstream discharge station operated by Dirección General de Aguas^45^. This discharge station is located 14 km downstream from Lago Grey (Fig. 1a). The watershed of Río Grey at the station has an area of 918 km^2^, which includes Glaciar Grey, Glaciar Pingo, and a part of Glaciar Tyndall^46^. Daily discharge data from 1981 onward are available at the Center for Climate and Resilience Research website (http://explorador.cr2.cl/).

## Supplementary information

Peer Review File

## Data Availability

The lake and meteorological data presented in the paper are deposited and available at Mendeley Data^47^ (10.17632/nxsd8n3tk6.1). The Landsat images are distributed at http://earthexplorer.usgs.gov/.
