# Supplementary material for: Subglacial discharge controls seasonal variations in the thermal structure of a glacial lake in Patagonia
Source: Nat Commun. 2021 Nov 2;12:6301. doi: 10.1038/s41467-021-26578-0 (PMC8563722; doi:10.1038/s41467-021-26578-0)
Supplement: Supplementary file 1 — Peer Review File [file 41467_2021_26578_MOESM1_ESM.pdf]

nature portfolio

Peer Review File

Subglacial discharge controls seasonal variations in the thermal structure of a glacial lake in Patagonia

Open Access This file is licensed under a Creative Commons Attribution 4.0 International License, which permits use, sharing, adaptation, distribution and reproduction in any medium or format, as long as you give appropriate credit to the original author(s) and the source, provide a link to the Creative Commons license, and indicate if changes were made. In the cases where the authors are anonymous, such as is the case for the reports of anonymous peer reviewers, author attribution should be to 'Anonymous Referee' followed by a clear attribution to the source work. The images or other third party material in this file are included in the article's Creative Commons license, unless indicated otherwise in a credit line to the material. If material is not included in the article's Creative Commons license and your intended use is not permitted by statutory regulation or exceeds the permitted use, you will need to obtain permission directly from the copyright holder. To view a copy of this license, visit http://creativecommons.org/licenses/by/4.0/.

REVIEWER COMMENTS

Reviewer #1 (Remarks to the Author):

SUMMARY
This manuscript by Sugiyama et al. presents a 20 month-long record of water temperatures and currents from a mooring situated in a proglacial lake in Patagonia. These data are supported with sonar of the lake bed, meteorological and fluvial discharge records, and discrete measurements of lake temperature and turbidity at a range of depths for 3 additional sites within the lake. The data are used to develop a schematic model illustrating seasonal variation in the thermal structure and circulation of the lake. The research identifies a distinct seasonal cycle in these properties, specifically that the lake is isothermal in late winter, but diverges in summer into a relatively warm upper layer and a cold, turbid lower layer which increases in thickness until mid-summer. The thermocline between these layers corresponds closely to the elevation of a sill located within the lake. These seasonal variations are attributed to increased solar radiation in the summer months and the onset of subglacial discharge during the melt season. The results demonstrate that bathymetry and subglacial discharge are key controls on the thermal structure and circulation of glacial lakes, with the subglacial discharge particularly important for driving seasonal changes. The authors highlight the significance of these results for subaqueous melting and calving at the ice-margin, and lake ecosystems.

Enhanced mass loss at the margins of lake terminating glaciers has been increasingly recognised in the literature in recent years but, as the authors rightly assert, very little is known of glacial lake temperatures and circulation despite the increased prevalence of glacial lakes in recent decades. This research is particularly noteworthy and novel because it presents the first complete seasonal record of glacial lake temperatures and currents, and identifies some of the major controls on these variables. The methods employed to acquire these data are sound, and the interpretation of the results is convincing and well-supported. The clarity of the writing and presentation of the results are very good (although I have highlighted a few very minor ways below in which these could be further strengthened). The advances presented here will be particularly valuable to the glacial research community because they increase understanding of ice-marginal processes and can be used to inform estimates of seasonal variability in subaqueous melt and calving (and thus mass loss) from other lake terminating glaciers.

Overall I found this to be a very strong and informative manuscript. My comments below are all relatively minor in nature.

MAIN COMMENTS
1. Because of the variety of sensors, variables and data collection sites, it took me a few attempts to fully comprehend the range of methodologies employed (particularly because there are two sources of lake temperature data and the supporting figures include numerous components). Adjusting the subheadings in the methods section could help to differentiate more clearly between the different methodologies used (and their associated objectives), and speed up interpretation of the figures. For example:
• Seasonal temperature and current measurement (Mooring)
• Discrete temperature, turbidity and depth measurement (GC 1-3/Casting/Depth Profiling)
Also, because the sonar sounding informed the location of the mooring, I feel that the sonar text (lines 293-299) should logically form the first paragraph of the methods section.

2. A sentence should also be added to the methods section to state the specific nature of the correlation coefficient presented in the results (lines 149-156) (e.g. Spearman's/Pearson's etc.). For future reference a Multivariate Linear Regression would be a more powerful method of analysing the Figure 5 data, because it can account for the additive effects of variables (e.g. wind and temperature on current), and would also determine the presence or absence of a significant difference between the currents at 58 m and 392 m depth. However, I do not think that there is a need to re-conduct the

statistical analyses here, because the data in Figure 5 only form a small element of the results and subsequent interpretation.

MINOR COMMENTS

Line 30: I presume that the floating terminus is inferred? This doesn't appear to be addressed specifically in the manuscript. It could also be useful to include a sentence in the abstract outlining the implications of this research - i.e. that subglacial discharge and bathymetry will dictate rates and mechanisms of mass loss for lake terminating glaciers (if space is available).

Line 34: Should 'calving' be changed to 'water-terminating'? Otherwise there is an element of circular reasoning in the sentence (i.e. calving glaciers are affected by underwater melting, which makes them calve).

Lines 39-40: Although 'other regions' are noted, I think it would be useful to specifically include the Himalaya in this list due the magnitude and prominence of mass loss at lake-terminating glaciers in the region, and its associated implications (e.g. King et al. 2019, Glacial lakes exacerbate Himalayan glacier mass loss, Scientific Reports).

Lines 47-48: I think this sentence could be amended to improve clarity. For example changing '…are significantly different from those in glacial fjords' to '…is significantly different to that in glacial fjords' (or similar).

Lines 59-63: These sentences should be written in the past tense because they refer to the research already completed in ref. 26.

Line 64: If space is available, I feel that this implication could be expanded upon slightly (presumably that the nature of lake stratification can therefore dictate dominant calving mechanisms and rates?).

Line 66: Insert 'have' between 'studies' and 'measured'. Is it also worth citing refs. 19-27 again at the end of this sentence?

Lines 83-84 & 86-87: Could these two sentences be combined to minimise repetition?

Lines 89-90: Although cited to ref. 27, the phrasing of this sentence suggests that the reported bathymetry is associated with ref. 29. Perhaps changing '…but lake bathymetry was reported only for…' to '…but to date lake bathymetry has only been reported for…' (or similar) would help emphasise that there were two separate studies?

Line 120: add 'Figure 3a' after 'Lago Grey'.

Line 126: '391 m' should read '392 m'.

Line 129: '…temperature then increased in late January…' - this is not particularly evident in Figures 3b or 6g. If anything, cooling appears to be the predominant trend in late January at 223-392 m.

Lines 154-156: I have three thoughts on this sentence. Firstly, that it is primarily interpretation and would therefore be more appropriate in the discussion section, rather than the results. Secondly, that in isolation the statistics in Figure 5 cannot robustly support this interpretation (i.e. this interpretation is also reliant on the other data presented in the manuscript and existing knowledge of the relationship between air temperature and subglacial discharge - hence why this would be better explored in the discussion). Finally, did the authors consider investigating the relationship between current and lake temperature in the statistics?

Line 167: If the data in this manuscript are the first seasonal records of glacial lake thermal structure

and circulation in Patagonia I am not sure they can be described as 'typical' (surely we can't know this until there are similar seasonal records from other lakes?).

Line 193: Add (193 m) after 'second sensor'.

Lines 230-237: This section of text may be more useful directly following the sentence ending in '…139 m' (on line 203). Personally, I found myself asking why subglacial discharge would affect the mid-depths more after mid-summer on line 203, but had to wait for the explanation.

Lines 253-256: Although it is implied here, I think it could also be worth emphasising that lake bathymetry is therefore likely to be a key control on dominant calving mechanisms. Presumably, calving losses in shallow lakes and/or those without a sill are likely to be dominated by frequent melt-undercut driven calving events, whereas calving losses in deeper lakes with a sill will predominantly occur via lower frequency, but higher magnitude, buoyancy driven calving events. Knowledge of lake bathymetry is therefore critical to understanding freshwater calving processes (and mass loss) at the local scale - this is certainly one of my take-home messages!

Lines 264-265: Is this statement a working hypothesis, or a reference to existing research (in which case it is missing a citation)?

FIGURE COMMENTS
Figure 1: I think there is a typo in the UTM-easting axis above (a) - there are two 620s!

Figure 2: I think a text label indicating the mooring would facilitate interpretation of (e). In the accompanying caption, should '3c' (line 489) read '3b'? Also on line 489, should '4(i)-(l)' read '6(e)-(h)'?

Figure 3: A horizontal line denoting the elevation of the sill could help with interpretation of Figure 3d (particularly to illustrate the thermocline exceeding/overtopping the sill elevation in Jan 2018). Noting that the water temperatures in (b) and currents in (c) were recorded at the mooring in the accompanying captions (lines 498 and 499 respectively) could also assist interpretation of the figure (particularly when the figures are consulted prior to reading the methods and/or results).

Figure 4: There is a minor inconsistency in the dates used in the figure and the figure caption. The second column of the figure states 'Oct-Nov 2017', but the text in the caption below (line 510) states 'October-December 2017'.

Figure 5: What does 'v.s.' signify on line 518?

Figure 6: Oscillation is spelt incorrectly in Figure 6f.

Figure 7: Because the minimal vertical thermal gradient is only reached in Sept (Figs. 2d and 3d), would 'Late winter' or 'Early spring' be a more representative label for (a) and the supporting caption? There appears to be a considerable lag between atmospheric and lake cooling in Figure 3!

Reviewer #2 (Remarks to the Author):

Overview
This is an excellent paper which gives a detailed yet succinct insight into a major study on a large glacial lake system in contact with a glacier in Patagonia that has experienced large calving events in the 1990s. Undertaking such a study on a large proglacial lake system and attaining high quality year round data (namely water temperature profile, turbidity and current data) is a major development in

assessing the evolution of proglacial lake thermal regime and influence on glacier retreat rates.

Key Results and Significance

Firstly, the paper reports the persistence of a warm layer (>4oC) above 100m depth persisting for January, February and March 2017. This is the first report of a warm surface layer persisting through the melt season (a very important result) at an ice contact proglacial lake, which potentially has important implications for subaqueous melt rates at the ice front. Although the sensors are too far from the ice front to confirm whether water at this temperature was consistently in contact with the ice. Previous observations of thermal notch incision and the presence of an underwater ice foot reported in Sugiyama et al., (2019) suggest that warm water contact with the glacier terminus may be limited to the near surface layers. This remains an important result as the thermal notch formation would promote subaerial calving and this is central to their argument that if subaerial terminus retreat exceeds subaqueous retreat rates, then an ice foot will protrude subaqueously into the lake from the glacier front.

The presence of a protruding ice foot into the lake would be further sustained by the cold bottom layers that they report below 100m. Critically the large volume of subaqueous ice protruding into the lake would subject the glacier to higher forces of buoyancy, which combined with removal of subaerial ice likely lead to the flotation of the terminus. Therefore likely explaining the large calving event(s) and rapid retreat of the glacier during the 1990s. The persistence of cold turbid glacial meltwater (and limited down mixing of warm layers) reported in this study is central to this process and the lack of upwelling plumes is also an important finding, as their absence helps to maintain the stratified water column and potentially unbalanced glacier geometry that has led to periods of rapid retreat. Crucially, the injection of less turbid glacial meltwater later in summer would potentially destabilise this stratification, if suspended sediment concentration was low enough for the meltwater to be more buoyant than water at the temperature density maximum (3.98oC), which could in theory lead to wider scale mixing of surface and deeper waters. The results presented show that this is not the case and that the warmer shallower layers persist throughout the melt season, although there is a weakening of the thermocline and some mixing between 100 to 200m depth.

Validity, Data and Methodology

The data and methodology are of the highest standard. The validity of the results and argument in the discussion is high, although some comments could be further supported by evidence presented by this study and Sugiyama et al. (2019). Ideally the maximum depth of wind driven mixing would be constrained, however this is difficult to achieve given the interlinkages in the system.

Analytical Approach

Analytical Approach is of the highest standard.

Suggested Improvements

(Line number and comment)

52/53 I think a reference to the wind driven circulation reported in Chikita et al., (1999) should be acknowledged here. Does this not count as active circulation or is this just referring to internal lake forcing?

53/54 Define relatively cold (<4C). Also I think Roehl (2006) needs to be cited here, as she found warmer temperatures directly at the ice front and calculated substantial melt rates.

62/63 mention thermal notch/undercut profile

64/65 Add an extra bit of explanation here for non-specialists ‘as subaerial retreat exceeded most subaqueous retreat, so a greater proportion of the ice front was submerged without subaerial mass countering the buoyancy forces acting on the submerged mass’.

90 The bathymetry data presented here is really important, it seems to be previously unpublished and I think more should be made of it’s importance as it’s crucial to determining the thermal regime and geometry evolution of the ice front.

94 How close was GC1 to the ice front? Looks like it was ~100m, which is pretty close and should be stated. ‘Between 100m to 1.8km of the ice front…’

122 Give more details about the magnitude of this drop (‘from ~4oC to 2.1oC) and how many hours. This is a really substantial drop and the magnitude of it strongly supports the assertion that it is due to input of meltwater at 139m rather than wind driven mixing.
156 What was the directional data from the current sensor at 139m? I wonder if this varied depending on depth of wind driven mixing (presumably correlating with wind speed) against input of meltwater?
178 You could potentially mention the air temperature at the AWS here to give a bit of an indication of how much of the ablation area was subject to the rainfall event. Although to be honest I think the discharge data speaks for itself and I agree that rainfall events can have a large impact on proglacial lake thermal regime.
190 I think it’s really difficult to distinguish whether some of these oscillations at 139m depth are wind driven or meltwater driven. Between mid-December to mid-January it looks to me (from Figure 6) that peaks in wind speed are slightly in front of peaks in temperature at 139m, suggesting downward mixing of the warmer surface layers. I think this needs to be noted at this point too.
204 Was it particularly gradual? Presumably this would depend on if outflow over the sill was equal to input from glacial discharge (the latter may have exceeded the former for periods and led to the turbulent oscillations across the thermocline?). It looks like there is periodic mixing between the two across the thermocline from mid-December to mid-January. Then there is the dramatic cooling event at 139m, with short term spike straight after, and then persisitently low temperatures after mid-January at 139m depth.
214 Similar to point above. I think density of the meltwater is likely to be important here; as you have argued elsewhere, if the suspended sediment concentration was lower then the newer meltwater would sit higher in the water column. Again there is the question of how much turbid water was escaping over the sill.
234 Maybe add something onto the end of the sentence like ‘resulting in meltwater with a lower density than earlier in the summer’.
241 The importance of the summer warm layer persisting for several months should be highlighted. To my knowledge this is the first time that this has been constrained through the melt season.
249 I think a little more needs to be added here to explain to the none specialist how preservation of subaqueous ice due to colder temperatures and removal of subaerial ice unbalances the ice front.
253 I agree that buoyancy forces were likely to be a strong driving factor of the rapid retreat/break up, particularly if there was a large volume of subaqueous ice that didn’t have subaerial ice above it. I think if the terminus achieved flotation then there would be a distinctive crevasse pattern (particularly at the hinge point) with this (as found in Tsutaki et al., 2013), was this visible in the Landsat imagery? Also Boyce et al 2007 reported buoyancy driven calving event at Mendenhall.
256 Yes I agree. Although I think you need to re-jig this paragraph to make the argument more clearly as per the comments above.
262 Tiberti, R., Buscaglia, F., Callieri, C., Rogora, M., Tartari, G. and Sommaruga, R., 2020. Food web complexity of high mountain lakes is largely affected by glacial retreat. Ecosystems, 23(5), pp.1093-1106.
416 Rivera

Clarity and Context

Generally excellent. One paragraph could do with altering slightly to make the argument clearer for non-specialists (see improvements).

References

Tiberti, R., Buscaglia, F., Callieri, C., Rogora, M., Tartari, G. and Sommaruga, R., 2020. Food web complexity of high mountain lakes is largely affected by glacial retreat. Ecosystems, 23(5), pp.1093-1106.

Röhl, K., 2006. Thermo-erosional notch development at fresh-water-calving Tasman Glacier, New Zealand. Journal of Glaciology, 52(177), pp.203-213.

Reviewer Expertise

PhD in Proglacial Lake Thermal Regime.

****

Reviewer #1

MAIN COMMENTS

1. Because of the variety of sensors, variables and data collection sites, it took me a few attempts to fully comprehend the range of methodologies employed (particularly because there are two sources of lake temperature data and the supporting figures include numerous components). Adjusting the subheadings in the methods section could help to differentiate more clearly between the different methodologies used (and their associated objectives), and speed up interpretation of the figures. For example:

• Seasonal temperature and current measurement (Mooring)
• Discrete temperature, turbidity and depth measurement (GC 1-3/Casting/Depth Profiling)

Also, because the sonar sounding informed the location of the mooring, I feel that the sonar text (lines 293-299) should logically form the first paragraph of the methods section.

We corrected the subheadings and moved the text on sonar measurement to the top of the Method section. Please see the changes in Line 286–310.

2. A sentence should also be added to the methods section to state the specific nature of the correlation coefficient presented in the results (lines 149-156) (e.g. Spearman's/Pearson's etc.). For future reference a Multivariate Linear Regression would be a more powerful method of analysing the Figure 5 data, because it can account for the additive effects of variables (e.g. wind and temperature on current), and would also determine the presence or absence of a significant difference between the currents at 58 m and 392 m depth. However, I do not think that there is a need to re-conduct the statistical analyses here, because the data in Figure 5 only form a small element of the results and subsequent interpretation.

The information (Pearson correlation coefficient) is added in the main text (Line 155) and in the figure caption (Line 548). I believe a multivariate regression analysis is not appropriate here, especially because of the indirect cause and effect relationship between air temperature and current at 392 m.

MINOR COMMENTS

Line 30: I presume that the floating terminus is inferred? This doesn't appear to be addressed specifically in the manuscript. It could also be useful to include a sentence in the abstract outlining the implications of this research - i.e. that subglacial discharge and bathymetry will dictate rates

and mechanisms of mass loss for lake terminating glaciers (if space is available).

I understand that the comment "floating terminus is inferred" refers to Glaciar Grey. The answer is "No". Relatively steep surface slope near the ice front suggests grounded terminus, but we do not have evidence to exclude floatation. This last sentence gives a general implication of the study, thus we leave it as it was. The suggested sentence is included as the second last sentence of the abstract (Line 28–30).

Line 34: Should ‘calving’ be changed to ‘water-terminating’? Otherwise there is an element of circular reasoning in the sentence (i.e. calving glaciers are affected by underwater melting, which makes them calve).

The sentence is revised as suggested (Line 34).

Lines 39-40: Although ‘other regions’ are noted, I think it would be useful to specifically include the Himalaya in this list due the magnitude and prominence of mass loss at lake-terminating glaciers in the region, and its associated implications (e.g. King et al. 2019, Glacial lakes exacerbate Himalayan glacier mass loss, Scientific Reports).

I agree with the comment. "the Himalayas" is included with the suggested reference (Line 40).

Lines 47-48: I think this sentence could be amended to improve clarity. For example changing ‘…are significantly different from those in glacial fjords’ to ‘…is significantly different to that in glacial fjords’ (or similar).

I split the texts on "water circulation" and "thermal regime" here and several sentences later. I hope clarity is improved by the changes in Line 47–48 and 52–53.

Lines 59-63: These sentences should be written in the past tense because they refer to the research already completed in ref. 26.

Actually, "stratification prohibits vertical mixing" and "surface water is distributed…" are general facts and not specifically referred to ref. 26. Thus, we leave the tense of the sentence as it was (Line 59–61).

Line 64: If space is available, I feel that this implication could be expanded upon slightly

(presumably that the nature of lake stratification can therefore dictate dominant calving mechanisms and rates?).

By also including suggestions from Reviewer 2, I revised the final part of this paragraph. Please see Line 62–68.

Line 66: Insert 'have' between 'studies' and 'measured'. Is it also worth citing refs. 19-27 again at the end of this sentence?

Corrected as suggested (Line 69).

Lines 83-84 & 86-87: Could these two sentences be combined to minimise repetition?

By considering the comment, I modified the order and construction of the sentences (Line 86–94).

Lines 89-90: Although cited to ref. 27, the phrasing of this sentence suggests that the reported bathymetry is associated with ref. 29. Perhaps changing '…but lake bathymetry was reported only for…' to '…but to date lake bathymetry has only been reported for…' (or similar) would help emphasise that there were two separate studies?

Corrected as suggested (Line 90–91).

Line 120: add 'Figure 3a' after 'Lago Grey'.

I assume the reviewer suggest to add "Figure 1a" to show the location of the discharge station. This is done (Line 124).

Line 126: '391 m' should read '392 m'.

Thank you for pointing out this error (Line 130).

Line 129: '…temperature then increased in late January…' - this is not particularly evident in Figures 3b or 6g. If anything, cooling appears to be the predominant trend in late January at 223-392 m.

Closer look of Figure 3b shows temperature at 223–392 m turned to increase in late January. The change in the trend is also evident in Figure 3d. I revised the manuscript to "…until temperature then began increase…" (Line 133).

Lines 154-156: I have three thoughts on this sentence. Firstly, that it is primarily interpretation and would therefore be more appropriate in the discussion section, rather than the results. Secondly, that in isolation the statistics in Figure 5 cannot robustly support this interpretation (i.e. this interpretation is also reliant on the other data presented in the manuscript and existing knowledge of the relationship between air temperature and subglacial discharge - hence why this would be better explored in the discussion). Finally, did the authors consider investigating the relationship between current and lake temperature in the statistics?

I agree with the first two points. Firstly, the sentence mentioned above is modified to avoid data interpretation in Result section (Line 158–160). The link between wind and near-surface currents is explained later in the discussion on the October observation (Line 185–188). The mechanism connecting air temperature and currents in deep water is discussed with the December data (Line 196–200). I believe the concern of the reviewer is addressed by these changes.
Finally, we did not compare current and lake temperature because no correlation is observed in the data. One of the reason of the lack in correlation is that temperature changes much slower than current. This is very clear if we compare temperature at 392 m (black line in Figs 6e–h) and current at the same depth (black line in Figs 6i–l). The same is true at 58 m (blue lines in Figs 6e–h and Figs 6i–l). Considering the difference in the two physical properties, it is reasonable to find no correlation between temperature and current. Anyway, thank you for this comment. It was good for me to revisit this point.

Line 167: If the data in this manuscript are the first seasonal records of glacial lake thermal structure and circulation in Patagonia I am not sure they can be described as 'typical' (surely we can't know this until there are similar seasonal records from other lakes?).

I agree with the comment and removed "that are typical" (Line 169).

Line 193: Add (193 m) after 'second sensor'.

Added (Line 201). It's "139 m".

Lines 230-237: This section of text may be more useful directly following the sentence ending in

'…139 m' (on line 203). Personally, I found myself asking why subglacial discharge would affect the mid-depths more after mid-summer on line 203, but had to wait for the explanation.

I appreciate this comment. However, we prefer to leave this section here as a part of the overview of the seasonal variations. The influence of the discharge on the mid-depth layer already started in December (Line 189–191) and greater impact was observed later in January (Line 201–206). Our intention is to provide brief interpretation based on data in "Interpretation of the data", and summarize the overview of the seasonal variations in the following subsection "Seasonal variations in lake thermal structure". I believe this summary subsection with illustrations (Figure 7) helps the reader to understand the seasonal changes in the lake conditions, including the influence of the discharge on the mid-depth layer later in summer.

Lines 253-256: Although it is implied here, I think it could also be worth emphasising that lake bathymetry is therefore likely to be a key control on dominant calving mechanisms. Presumably, calving losses in shallow lakes and/or those without a sill are likely to be dominated by frequent melt-undercut driven calving events, whereas calving losses in deeper lakes with a sill will predominantly occur via lower frequency, but higher magnitude, buoyancy driven calving events. Knowledge of lake bathymetry is therefore critical to understanding freshwater calving processes (and mass loss) at the local scale - this is certainly one of my take-home messages!

Thank you for this comment. The suggested text is very helpful to stress the importance of our data. Please see Line 266–271.

Lines 264-265: Is this statement a working hypothesis, or a reference to existing research (in which case it is missing a citation)?

This is a working hypothesis. "Presumably" is added to the the sentence (Line 279).

FIGURE COMMENTS

Figure 1: I think there is a typo in the UTM-easting axis above (a) - there are two 620s!

Thank you for pointing out this error. Corrected.

Figure 2: I think a text label indicating the mooring would facilitate interpretation of (e). In the accompanying caption, should '3c' (line 489) read '3b'? Also on line 489, should '4(i)-(l)' read '6(e)-(h)'?

A text label "Mooring sensors" is added to Figure 2e. I thank the reviewers for pointing out the errors in the text labels, which are corrected.

Figure 3: A horizontal line denoting the elevation of the sill could help with interpretation of Figure 3d (particularly to illustrate the thermocline exceeding/overtopping the sill elevation in Jan 2018). Noting that the water temperatures in (b) and currents in (c) were recorded at the mooring in the accompanying captions (lines 498 and 499 respectively) could also assist interpretation of the figure (particularly when the figures are consulted prior to reading the methods and/or results).

A dashed line is added to Figure 3d to show the depth of the sill. "by the mooring" is included in the sentences so that the data is Figs 3b and 3c are from the long-term measurement (Line 523 and 525).

Figure 4: There is a minor inconsistency in the dates used in the figure and the figure caption. The second column of the figure states ‘Oct-Nov 2017’, but the text in the caption below (line 510) states ‘October-December 2017’.

Thank you for pointing out this typo. The text in Figure 4 is corrected.

Figure 5: What does ‘v.s.’ signify on line 518?

It should have "vs.", which abbreviates versus. I correct it to "versus" (Line 545).

Figure 6: Oscillation is spelt incorrectly in Figure 6f.

Thank you, corrected.

Figure 7: Because the minimal vertical thermal gradient is only reached in Sept (Figs. 2d and 3d), would ‘Late winter’ or ‘Early spring’ be a more representative label for (a) and the supporting caption? There appears to be a considerable lag between atmospheric and lake cooling in Figure 3!

Yes, I agree. Figure 7a is labelled as "Late winter".

****

Reviewer #2 (Remarks to the Author):

Suggested Improvements

(Line number and comment)

52/53 I think a reference to the wind driven circulation reported in Chikita et al., (1999) should be acknowledged here. Does this not count as active circulation or is this just referring to internal lake forcing?

Here, we discuss active circulation driven by upwelling of subglacial meltwater discharge, thus mentioning wind driven near surface circulation is not adequate. The work by Chikita et al. (1999) is referred to as a key study in Himalaya (Line 54).

53/54 Define relatively cold (<4C). Also I think Roehl (2006) needs to be cited here, as she found warmer temperatures directly at the ice front and calculated substantial melt rates.

Unfortunately, it is difficult to define "relatively cold water" because of the diversity of the study region from Himalaya to Patagonia. Roehl (2006) studied details in water temperature near the ice front (< ~100 m) and reported melting at the water line. Here, we review lake thermal structures and circulations in a larger scale. Because of these reasons, we prefer to leave the text as it is.

62/63 mention thermal notch/undercut profile

Thermal notch is formed by melting at the waterline, which destabilize ice above the water. This is a process different from what we introduce here. The observation in Glaciar Grey revealed underwater melting at the depth 0–50 m, which causes protrusion of the deeper ice into the lake. Therefore, I believe mentioning "thermal notch" is not adequate here.

64/65 Add an extra bit of explanation here for non-specialists ‘as subaerial retreat exceeded most subaqueous retreat, so a greater proportion of the ice front was submerged without subaerial mass countering the buoyancy forces acting on the submerged mass’.

Following the suggestion from the reviewer, the last several sentences of this paragraph is revised (Line 62–68).

90 The bathymetry data presented here is really important, it seems to be previously unpublished and I think more should be made of it's importance as it's crucial to determining the thermal regime and geometry evolution of the ice front.

The text is revised to highlight the importance of the bathymetry data (Line 90–91).

94 How close was GC1 to the ice front? Looks like it was ~100m, which is pretty close and should be stated. 'Between 100m to 1.8km of the ice front…'

The text is revised as suggested (Line 98).

122 Give more details about the magnitude of this drop ('from ~4oC to 2.1oC) and how many hours. This is a really substantial drop and the magnitude of it strongly supports the assertion that it is due to input of meltwater at 139m rather than wind driven mixing.

It is difficult to give more details based on Figure 3b. The temperature change is not a simple drop within hours or so, but it consists of up and down over several days as illustrated in Figure 6b. Therefore, we leave the text as it is and explain the details of the temperature drop later in Discussion "Interpretation of the data" (Line 201–213).

156 What was the directional data from the current sensor at 139m? I wonder if this varied depending on depth of wind driven mixing (presumably correlating with wind speed) against input of meltwater?

Unfortunately, we do not have a current sensor at 139 m (only temperature was measured).

178 You could potentially mention the air temperature at the AWS here to give a bit of an indication of how much of the ablation area was subject to the rainfall event. Although to be honest I think the discharge data speaks for itself and I agree that rainfall events can have a large impact on proglacial lake thermal regime.

This is a good suggestion. Based on air temperature (~5–10°C), a large area of the glacier was affected by the rain. This point is mentioned (Line 179–180).

190 I think it's really difficult to distinguish whether some of these oscillations at 139m depth are wind driven or meltwater driven. Between mid-December to mid-January it looks to me (from

Figure 6) that peaks in wind speed are slightly in front of peaks in temperature at 139m, suggesting downward mixing of the warmer surface layers. I think this needs to be noted at this point too.

I do not believe the temperature change at 139 m is caused by wind driven mixing, because temperature at 58 m is stable during most of the oscillations at 139 m (Figs. 6f and g). We expect greater fluctuations at 58 m if mixing occurs from the surface. Air temperature and wind speed are very well correlated. We attribute the water temperature change at 139 m to warm air temperature (melt production) rather than high wind speed (Figs 6b and c).

204 Was it particularly gradual? Presumably this would depend on if outflow over the sill was equal to input from glacial discharge (the latter may have exceeded the former for periods and led to the turbulent oscillations across the thermocline?). It looks like there is periodic mixing between the two across the thermocline from mid-December to mid-January. Then there is the dramatic cooling event at 139m, with short term spike straight after, and then persisitently low temperatures after mid-January at 139m depth.

The upward migration of the thermocline from October to January is clearly observed in Figure 6d. The high frequency temperature change at 139 m described by the reviewer was occurring above the thermocline. To avoid misunderstanding, temperature of the cold bottom layer is defined as < ~2.5°C (Line 212).

214 Similar to point above. I think density of the meltwater is likely to be important here; as you have argued elsewhere, if the suspended sediment concentration was lower then the newer meltwater would sit higher in the water column. Again there is the question of how much turbid water was escaping over the sill.

I understand the point of the reviewer. However, seasonal turbidity variation is not available and turbid water overtopping the sill is difficult to quantify. Therefore, in the next subsection "Seasonal variations in lake thermal structure" (Line 224–246), we use information from this and previous studies to propose processes driving the seasonal change in the thermal regime observed in Lago Grey.

234 Maybe add something onto the end of the sentence like ‘resulting in meltwater with a lower density than earlier in the summer’.

A sentence is added to address the comment (Line 242–243).

241 The importance of the summer warm layer persisting for several months should be highlighted. To my knowledge this is the first time that this has been constrained through the melt season.

A sentence is added to highlight the importance of this observation (Line 253–254). Thank you for pointing out this.

249 I think a little more needs to be added here to explain to the none specialist how preservation of subaqueous ice due to colder temperatures and removal of subaerial ice unbalances the ice front.

The text is revised to explain the process envisaged here (Line 258–261).

253 I agree that buoyancy forces were likely to be a strong driving factor of the rapid retreat/break up, particularly if there was a large volume of subaqueous ice that didn't have subaerial ice above it. I think if the terminus achieved flotation then there would be a distinctive crevasse pattern (particularly at the hinge point) with this (as found in Tsutaki et al., 2013), was this visible in the Landsat imagery? Also Boyce et al 2007 reported buoyancy driven calving event at Mendenhall.

I understand that the reviewer is mentioning the breakup in 1990s. Unfortunately, not sufficiently good quality and quantity of satellite images are available during the event (not many satellites 30 years ago and the region is mostly covered by cloud).

256 Yes I agree. Although I think you need to re-jig this paragraph to make the argument more clearly as per the comments above.

The paragraph is revised. Please see the change (Line 258–271).

262 Tiberti, R., Buscaglia, F., Callieri, C., Rogora, M., Tartari, G. and Sommaruga, R., 2020. Food web complexity of high mountain lakes is largely affected by glacial retreat. Ecosystems, 23(5), pp.1093-1106.

The suggested paper studied lakes fed by glacial streams, while mentioning here is a lake fed directly by calving glaciers. I include the suggested citation in the literatures referred to in the final sentence of this paragraph (Line 283).

REVIEWERS' COMMENTS

Reviewer #1 (Remarks to the Author):

The responses and revisions in this updated manuscript by Sugiyama et al. are thorough and persuasively address all the main and minor comments raised in my original review. I believe these revisions have further improved an already strong manuscript, particularly the increased detail regarding the interpretation of the processes, and the increased emphasis on the broader implications for mass loss and calving mechanisms. By advancing knowledge of ice-marginal processes and controls on mass loss at lake-terminating glaciers this manuscript, and the novel data set it presents, will be particularly valuable to the glacial research community.

Reviewer #2 (Remarks to the Author):

Overview
This is an excellent paper which gives a detailed yet succinct insight into a major study on a large glacial lake system in contact with a glacier in Patagonia that has experienced large calving events in the 1990s. Undertaking such a study on a large proglacial lake system and attaining high quality year round data (namely water temperature profile, turbidity and current data) is a major development in assessing the evolution of proglacial lake thermal regime and influence on glacier retreat rates. The full importance of this is now fully acknowledged in the revised manuscript and the authors have comprehensively responded to all of the reviewers comments after careful consideration. The revised manuscript has been thoroughly improved in response to the reviewers comments and kindly acknowledged too (thank you!).

Key Results and Significance
Firstly, the paper reports the persistence of a warm layer (>4 C) above 100m depth persisting for January, February and March 2017. This is the first report of a warm surface layer persisting through the melt season (a very important result) at an ice contact proglacial lake, which potentially has important implications for subaqueous melt rates at the ice front. Although the sensors are too far from the ice front to confirm whether water at this temperature was consistently in contact with the ice. Previous observations of thermal notch incision and the presence of an underwater ice foot reported in Sugiyama et al., (2019) suggest that warm water contact with the glacier terminus may be limited to the 'near surface layers'. This remains an important result as the thermal notch formation would promote subaerial calving and this is central to their argument that if subaerial terminus retreat exceeds subaqueous retreat rates, then an ice foot will protrude subaqueously into the lake from the glacier front. Further to this, the authors argue that an imbalance in geometry across the subaqueous ice front can also be brought about by higher subaqueous melt rates in the upper (<100m depth) water column (rather than just the near-surface incision of thermal notches) resulting in higher melt rates and consequently deeper ice (>100m) protruding subaqueously at glacier Grey. This is also likely at other deep proglacial lakes and is a key result so further distinguishing between the two (a. thermal notch b. thermal subaqueous geometry imbalance) may be useful to inform other workers in the field and provide further stimulus to advance the science (particularly sonar surveys).

The presence of a protruding ice foot into the lake would be further sustained by the cold bottom layers that they report below 100m. Critically the large volume of subaqueous ice protruding into the lake would subject the glacier to higher forces of buoyancy, which combined with removal of subaerial ice likely lead to the flotation of the terminus. Therefore likely explaining the large calving event(s) and rapid retreat of the glacier during the 1990s, which has been communicated well in the revised manuscript. The persistence of cold turbid glacial meltwater (and limited down mixing of warm layers) reported in this study is central to this process and the lack of upwelling plumes (>100m away from the terminus) is also an important finding, as their absence helps to maintain the stratified water column and potentially unbalanced glacier geometry that has led to periods of rapid retreat. Crucially,

the injection of less turbid glacial meltwater later in summer would potentially destabilise this stratification, if suspended sediment concentration was low enough for the meltwater to be more buoyant than water at the temperature density maximum (3.98 C), which could in theory lead to wider scale mixing of surface and deeper waters. The results presented show that this appears not to be the case and that the warmer shallower layers persist throughout the melt season, with meltwater suspended sediment concentration being a controlling factor on the stability of stratification (I think the spikes in temperature in the deeper sensors are good evidence for this). Although there is a weakening of the thermocline and some fluctuations/mixing between 100 to 200m depth during March (which could be further explored in future work).

Validity, Data and Methodology
The data and methodology are of the highest standard. The validity of the results and argument in the discussion is high. Some comments have been further supported by evidence presented by this study and Sugiyama et al. (2019). Ideally the maximum depth of wind driven mixing would be constrained, however this is difficult to achieve given the inter-linkages in the system but could be included in future work.

Analytical Approach
Analytical Approach is of the highest standard and a couple of clarification points have been made in the revised manuscript.

Clarity and Context
Generally excellent. One paragraph has been altered slightly in the improvements and this makes the argument clearer for non-specialists.
